# Sensory reanimation of the hand by transfer of the superficial branch of the radial nerve to the median and ulnar nerve

**DOI:** 10.1002/brb3.578

**Published:** 2016-10-09

**Authors:** Thilo L. Schenck, Shenyu Lin, Jessica K. Stewart, Konstantin C. Koban, Michaela Aichler, Farid Rezaeian, Riccardo E. Giunta

**Affiliations:** ^1^ Hand Surgery, Plastic Surgery and Aesthetic SurgeryLudwig‐Maximilians‐University MunichMunichGermany; ^2^ Department of Obstetrics and GynecologyUniversity Hospital rechts der IsarTechnical University MunichMunichGermany; ^3^ Research Unit Analytical PathologyHelmholtzzentrum MünchenGerman Research Center for Environmental HealthMunichGermany; ^4^ Department of Plastic Surgery and Hand SurgeryUniversity Hospital ZurichZurichSwitzerland

**Keywords:** hand trauma, histomorphometric nerve analysis, nerve injury, nerve reconstruction, peripheral nerve transfer, reinnervation

## Abstract

**Background:**

It remains a surgical challenge to treat high‐grade nerve injuries of the upper extremity. Extra‐anatomic reconstructions through the transfer of peripheral nerves have gained clinical importance over the past decades. This contribution outlines the anatomic and histomorphometric basis for the transfer of the superficial branch of the radial nerve (SBRN) to the median nerve (MN) and the superficial branch of the ulnar nerve (SBUN).

**Methods:**

The SBRN, MN, and SBUN were identified in 15 specimens and the nerve transfer performed. A favorable site for coaptation was chosen and its location described using relevant anatomical landmarks. Histomorphometric characteristics of donor and target were compared to evaluate the chances of a clinical success.

**Results:**

A suitable location for dissecting the SBRN was identified prior to its first bifurcation. Coaptations were possible near the pronator quadratus muscle, approximately 22 cm distal to the lateral epicondyle of the humerus. The MN and SBUN had to be dissected interfasciculary over 82 ± 5.7 mm and 49 ± 5.5 mm, respectively. Histomorphometric analysis revealed sufficient donor‐to‐recipient axon ratios for both transfers and identified the SBRN as a suitable donor with high axon density.

**Conclusion:**

Our anatomic and histomorphometric results indicate that the SBRN is a suitable donor for the MN and SBUN at wrist level. The measurements show feasibility of this procedure and shall help in planning this sensory nerve transfer. High axon density in the SBRN identifies it or its branches an ideal candidate for sensory reanimation of fingers and thumbs.

## 1 Introduction

A major limitation to the function of the hand is the loss of its sensibility, which also significantly impairs a patient's quality of life. At the beginning of the 20th century extra‐anatomic nerve transfers were introduced to treat large nerve defects as well as nerve injuries located proximally on the upper limb (Harris & Low, 1903). Especially motor nerve transfers to restore intrinsic hand function have gained popularity (Brown, Yee, & Mackinnon, 2009; Viguie, Lu, Huang, Rengen, & Carlson, 1997). When performing a nerve transfer a healthy nerve (donor) is dissected and connected to the injured nerve (target) distally to its injury. There is a drawback regarding nerve transfers in that they create a secondary defect when harvesting the donor nerve. It has to be carefully determined if the possible gain of function outweighs the created defect. The median nerve, followed by the ulnar nerve is of highest importance when it comes to hand sensibility. Consequently, nerve transfers have been described which redirect branches from the dorsum to the palmar side of the hand (Harris, 1921). While sensory nerve transfers have most frequently been indicated in open injuries, the procedure has also been applied in patients suffering from burns or leprosy (Dvali & Mackinnon, 2003; Özkan, Özer, & Gülgönen, 2001). This study's aim was to evaluate the anatomic and histomorphometric basis for the transfer of the superficial branch of the radial nerve (SBRN) either to the median nerve (MN) or to the superficial branch of the ulnar nerve (SBUN). These transfers could restore the hand's function and quality of life of patients with impaired sensibility of the palmar aspect of the hand due to injuries of the ulnar or median nerve.

## 2 Material and Methods

### 2.1 Anatomic dissection

**Figure 1:**
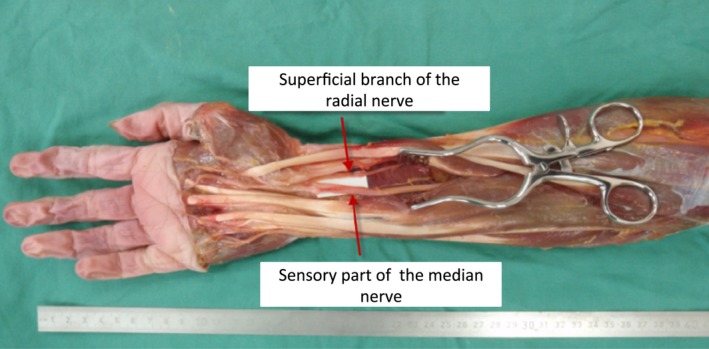
Transfer of the superficial branch of the radial nerve (SBRN) to the median nerve (MN). Transfer of the SBRN to the MN was performed in 15 fresh specimens. The SBRN was dissected proximally to its first bifurcation at the distal radial forearm. Mobilizing it in the proximal direction allowed a nerve transfer toward the MN. For maximum nerve length it was tunneled under the BR, FCR, and FPL muscle to reach the MN along its course. After exposing the MN and its thenar branch by opening the carpal tunnel, they were separated from each other until it was possible to perform a tension‐free coaptation of the MN and SBRN. The location of the coaptation, the bifurcation of the SBRN, the takeoff of the thenar branch, the length of neurolysis and the overall length of the forearms were measured in their relevance to the anatomic landmarks: lateral epicondyle of the humerus, styloid process of the radius, and pisiform bone (*n *= 15)

**Figure 2:**
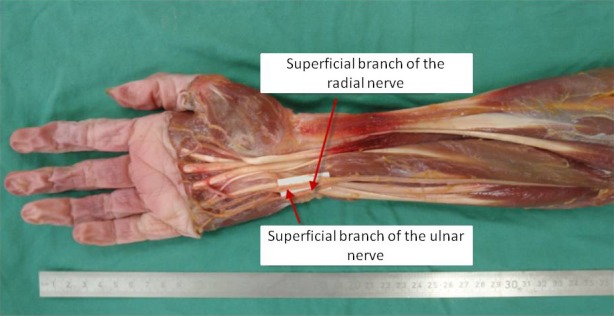
Transfer of the superficial branch of the radial nerve (SBRN) to the superficial branch of the ulnar nerve (SBUN). Transfer of the SBRN to the SBUN was performed in 15 fresh specimens. The SBRN was transferred as described in Fig. 1 but placed between the superficial and deep flexors to reach the SBUN along its course at the ulnar side of the wrist. The SBUN and DBUN were identified in the Guyon's canal and separated from each other in a retrograde manner until tension‐free coaptation of SBRN and SBUN could be achieved. In addition to the measurements described in Fig. 1, the location of the diversion of the SBUN and DBUN and the required length of neurolysis of SBUN and DBUN were described in their relevance to the previously mentioned anatomic landmarks. (*n *= 15)

We performed nerve transfers in 15 fresh anatomic specimens from donors without a history of neurological diseases. The SBRN was identified at the distal forearm and traced until its first bifurcation, prior to which it was transected. The SBRN was mobilized from its adjacent soft tissue from its bifurcation toward the elbow until it could be transposed to reach the ulnar and median nerve without relevant loss of length. The SBRN was tunneled under the brachioradialis muscle (BR), the flexor carpi radialis muscle (FCR) and the flexor pollicis longus muscle (FPL) for transposition toward the target nerves. To identify the sensory part of the MN, the carpal tunnel was opened and interfascicular neurolysis of the MN and its main motor component, the thenar branch, was performed until a tension‐free coaptation to the SBRN was possible (Fig. 1). The SBUN and its accompanying deep branch of the ulnar nerve (DBUN) were exposed in the Guyon's canal from where they were retrogradely separated from each other until it was possible to perform a tension‐free connection between the SBUN and the SBRN, which was placed between the superficial and deep flexors (Fig. 2). After performing the coaptations of the SBRN to either the MN or the DBUN the location of the coaptation, the bifurcation of the SBRN, the diversion of the SBUN and DBUN, the takeoff of the thenar branch and the overall length of the forearms were described in distance to suitable anatomic landmarks: the lateral epicondyle of the humerus, the styloid process of the radius and the pisiform bone.

### 2.2 Histomorphometry

**Figure 3:**
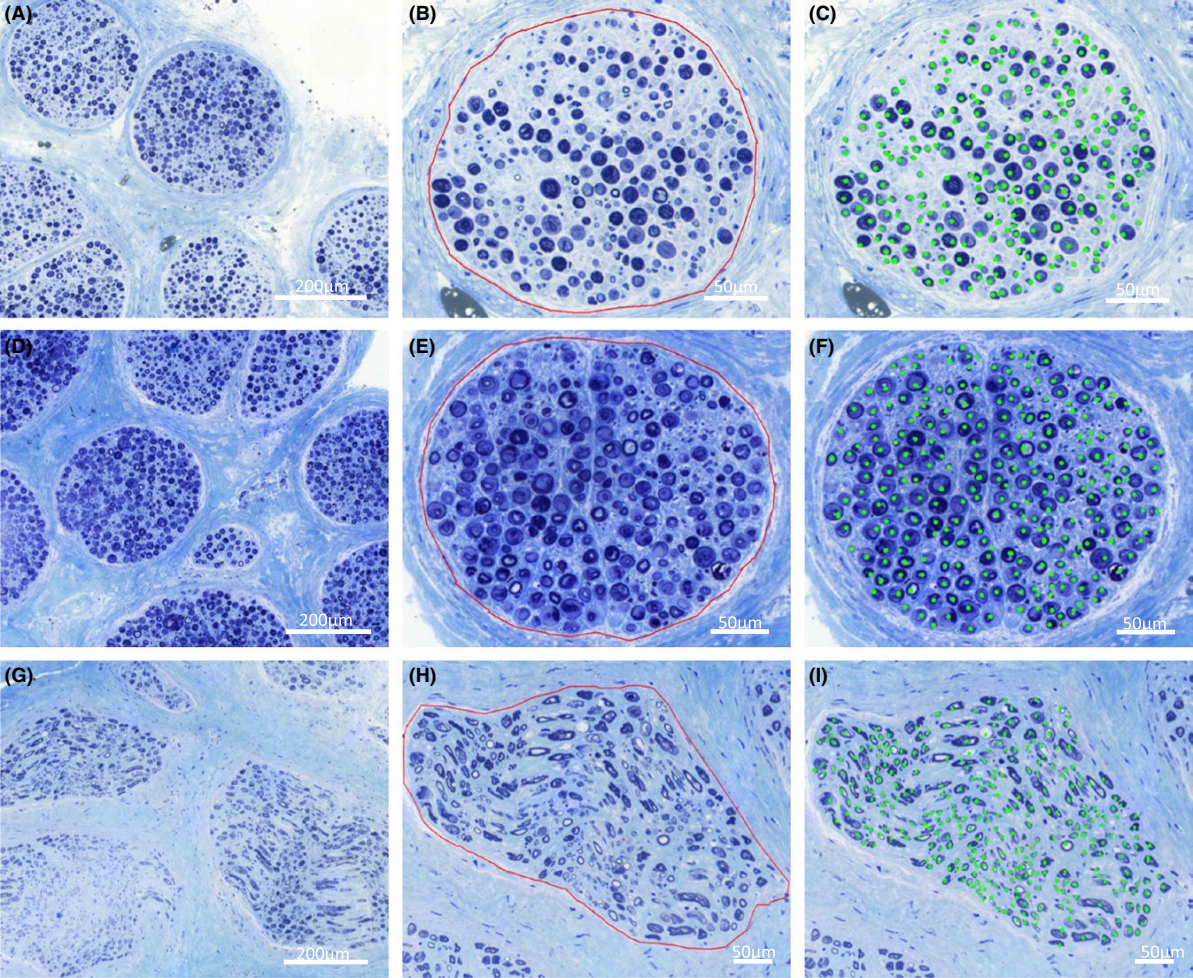
Histomorphometric analysis of superficial branch of the radial nerve (SBRN), median nerve (MN), and superficial branch of the ulnar nerve (SBUN). Samples from the SBRN (A, B, C), SBUN (D, E, F) and MN (G, H, I) were collected at the height of the coaptation. Samples were fixed in glutaraldehyde, embedded in epoxy raisin, cut to 1 μm semithin sections and stained with toluidine blue. At ×200 magnification, general nerve structure and fascicles were observed (A, D, G,). At ×600 magnification, cross‐sectional areas of individual fascicles were determined by a polygon approach (B, E, H) and axons were counted semiautomatically with a low cut‐off value for inclusion of 4 μm (C, F, I)

At the site of coaptation, nerve samples of 2–3 mm in length were excised from donor and target nerves. Samples were fixed over 60 min with 2.5% glutaradehayde in 0.1 mol/L sodium cacodylate buffer at 4°C (Science Services GmbH, Munich, Germany) and postfixed in a 2% aqueous osmium tetraoxide (Science Services GmbH). For dehydration an ascending alcohol series (30–100%) and propylene oxide were used before samples were epoxy raisin embedded (Merck, Darmstadt, Germany) and cured at 60°C for 24 hr. 1 μm semithin sections, right angled to the nerve orientation were cut with an ultramicrotome (Reichert Technologies, Munich, Germany), stained with 1% toluidine blue (Sigma‐Aldrich, Taufkirchen, Germany) and scanned at 20× magnification (Mirax Scannner, Carl Zeiss GmbH, Jena, Germany) (Fig. 3). At 600× magnification, axons were counted semiautomatically with a low cut‐off value for inclusion of 4 μm (ImageJ version 1.42; NIH, Bethesda, MD, USA). The cross‐sectional fascicle areas were measured by a software‐assisted polygon approach (Pannoramic Viewer 1.15; 3DHISTECH, Hungary). For each nerve the cross‐sectional surfaces of all fascicles was summed up to the total cross‐sectional fascicle area. Axon density was calculated as ratio of axon number and total fascicle area. To compare donor and target nerves, histomorphometric parameters were described as donor to target ratios. Statistical analysis consisted of a two‐tailed t‐test with *p *≤ .05 being considered as significant.

## 3 Results

### 3.1 Anatomic dissection

**Figure 4:**
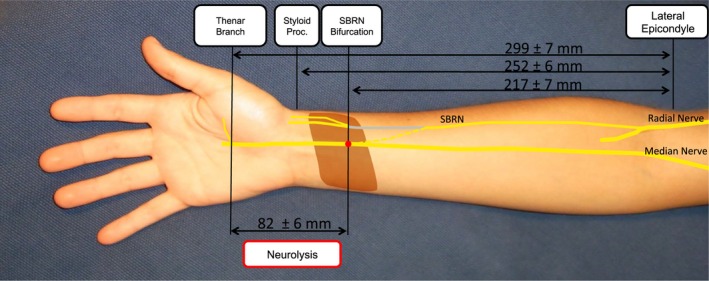
Transfer of the superficial branch of the radial nerve (SBRN) to the median nerve (MN). The SBRN was dissected proximal to its first bifurcation which was found 217 ± 7.1 mm distally to the lateral epicondyle of the humerus and 34.7 ± 5 mm proximal to the styloid process of the radius. For tension‐free coaptation, the MN had to be separated from the thenar branch over a distance of 82.1 ± 5.7 mm. The course of the SBRN before the transposition is shown in gray, whereas its course after transposition is an interrupted line and the coaptation is a red dot. Other highlighted structures are the pronator quadratus muscle (brown) and the pisiform bone (gray). For reasons of clarity, the radial nerve is shown just to the level, shortly beyond the takeoff of the SBRN. All data are presented as Mean ± *SEM*, (*n *= 15)

**Figure 5:**
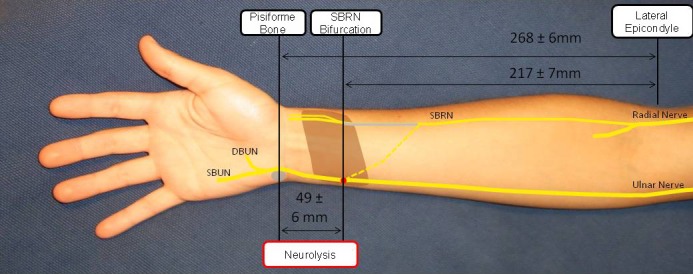
Transfer of the superficial branch of the radial nerve (SBRN) to the superficial branch of the ulnar nerve (SBUN). Harvesting of the SBRN is described in Fig. 4. For coaptation with the SBUN the SBRN was transposed to the ulnar side of the wrist. SBUN and DBUN were identified in the Guyon's canal, distally to the pisiform bone, which was found at 268 ± 6.0 mm distance to the lateral epicondyle. Starting at the Guyon's canal, SBUN and DBUN were separated over a length of 49.4 ± 5.5 mm to allow tension‐free coaptation. The course of the SBRN before the transposition is shown in gray. Its course after transposition is depicted by an interrupted line and the coaptation is represented with a red dot. Other highlighted structures are the pronator quadratus muscle (brown) and the pisiform bone (gray). For reasons of clarity, the radial nerve is shown just to the level shortly beyond the takeoff of the SBRN. All data are presented as Mean ± *SEM*, (*n *= 15)

In all specimens, the nerves of interest were identified without anatomic variations. The overall length of the forearms, measured from the lateral epicondyle of the humerus to the styloid process of the radius was 252 ± 6.3 mm. The SBRN was transected at its first bifurcation which was found 217 ± 7.1 mm distally to the lateral epicondyle of the humerus and 34.7 ± 5 mm proximal to the styloid process of the radius (Fig. 4). By separating the SBRN from its adjacent tissue over approximately 5–7 cm and by tunneling it under the BR, FCR and FPL muscle, maximum length was achieved and transposition towards the target nerves was possible without loss of length. For transfer to the SBUN, the SBRN was placed between the superficial and deep flexors to reach the ulnar aspect of the wrist. The thenar branch was separated from the MN over a distance of 82.1 ± 5.7 mm. The SBUN and the DBUN had to be separated over a length of 49.4 ± 5.5 mm beginning in the Guyon's canal whose associated landmark, the pisiform bone was located at 268 ± 6.0 mm distal to the lateral epicondyle (Fig. 5). The dorsal cutaneous branch of the ulnar nerve (DCBUN) which branches from the ulnar nerve approximately 7–9 cm proximal to the pisiform was not affected by this preparation in any forearm. The target nerves did not have to be transposed because mobilization of the SBRN was sufficient to reach them within their normal anatomic course. The height of the coaptation was defined by the maximum obtainable length of the SBRN, which reached 34.7 ± 5 mm proximal to the styloid process of the radius. At the coaptation a difference of calibers was noticeable. All data are given as the mean ± Standard Error of the Mean (*SEM*).

### 3.2 Histomorphometry

**Figure 6:**
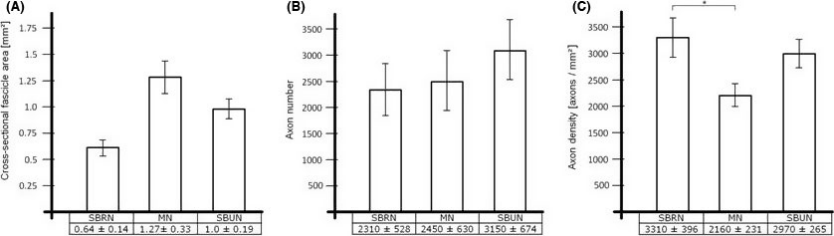
(A – C) We compared cross‐sectional fascicle areas (A), axon numbers (B) and axon densities (C) between donor (superficial branch of the radial nerve [SBRN], left columns) and targets (median nerve [MN], middle columns; superficial branch of the ulnar nerve [SBUN], right columns). Cross‐sectional fascicle areas and absolute axon numbers showed no significant differences. Axon density was highest in the SBRN, significantly exceeding the axon density of the median nerve. All data are presented as Mean ± *SEM*, (*n *= 10), (*p *< .05)

The total cross‐sectional fascicle areas were 0.64 ± 0.14 mm² for the SBRN**,** 1.27 ± 0.33 mm² for the MN and 1.0 ± 0.19 mm² for the SBUN (Fig. 6). The number of axons was 2310 ± 528 for the SBRN, 2450 ± 630 for the MN and 3150 ± 674 for the SBUN. No significant differences (*p* < .05) were found in comparison of donor to both targets in terms of cross‐sectional fascicle area and absolute axon numbers. The SBRN had the highest axon density (3310 ± 396), followed by the SBUN (2970 ± 265) and the MN (2160 ± 231) [all in axons/mm²]. Axon density of the SBRN was significantly higher than axon density of the median nerve (*p *< .05). All data are presented as Mean ± *SEM*, (*n *= 10).

## 4 Discussion

Microsurgical nerve transfers have become well‐established in the field of peripheral nerve surgery. They reduce reinnervation distance and time, allow restoration of function when the proximal nerve stump is unavailable and allow nerve surgery away from heavily destroyed or scarred tissues (Dvali & Mackinnon, 2003). Especially in the treatment of plexus injuries and facial paralysis, the introduction of nerve transfers and the essential postoperative care have brought new therapeutic options and helped to improve the patients’ quality of life (Belzberg, Dorsi, Storm, Moriarity, & Jerome, 2004; Klebuc, 2011; Kozin, 2008; Liu et al., 2012; Rodriguez et al., 2011; Schenck et al., 2011, 2015).

Loss of hand sensibility has a tremendous impact on a patient's working ability, social activities, and quality of life (Holdenried et al., 2013). Losing sensibility in the thumb alone is considered to impair 20% of hand function (Swanson, Hagert, & Swanson, 1983). Due to the obvious higher importance of palmar sensibility, reports of dorsal to palmar nerve transfers were among the earliest nerve transfers to be described. In his 1921 publication on the treatment of nerve injuries of First World War soldiers, R.I. Harris, the father of peripheral nerve transfers, reported successful a transfer of the SBRN to the MN (Harris, 1921). Since then, a modest number of sensory nerve transfers, including variations of dorsal to palmar nerve transfers and heterodigital nerve transfers have been reported (Bertelli, 2012; Brunelli, 2004; Murphy, Ray, & Mackinnon, 2012; Oberlin, Teboul, Severin, & Beaulieu, 2003; Turnbull, 1948). Up to now, we lack reports of large clinical series of sensory nerve transfers. For heterodigital nerve transfers success rates ranging from 72% to 85% have been reported (Özkan et al., 2001; Stocks, Cobb, & Lewis, 1991). Özkan and colleagues reported a two‐point discrimination of <10 mm in 15 of 25 patients with mainly heterodigital digital transfers (Özkan et al., 2001). Bertelli presented a series of eight cases where cutaneous branches of the MN were successfully transferred to the ulnar digital nerve of the small finger (Bertelli, 2012).

To perform successful motor nerve transfers, timing of the operation is a key as denervated muscle fibers are degraded and replaced by fibrotic tissue (Carlson, Borisov, Dedkov, Dow, & Kostrominova, 2002; Viguie et al., 1997). Therefore, motor transfers should only be considered if there is a chance for reinnervation within 1**–**2 years (Brown, Shah, & Mackinnon, 2009; Gordon, Sulaiman, & Boyd, 2003). For sensory transfers, timing of the operation is considered less critical (Brown, Shah, et al., 2009). For sensory transfers successful reinnervation has been described for up to 20 years after injury (Özkan et al., 2001). These long‐term results could be attributed to long survival of mechanoreceptors after axotomy as it was described for Pacinian corpuscles (Zelená, 1982).

The SBRN was harvested prior to its first bifurcation to maximize axon number and subsequently improve donor to target histomorphometric ratios. Mobilization of the SBRN and passing it under the BR, FCR, and FPL muscle helps to prevent the creation of a hypomochlion and allows a transposition to the target nerves within their normal anatomic course and without loss of length. Accordingly, the height of the coaptation is defined by the height of the SBRN bifurcation. At wrist level, the target nerves are mixed sensory and motor nerves, whereas the SBRN is purely sensory. To avoid a mismatching of motor and sensory axon, target nerves have to be separated from their accompanying motor parts at the carpus, where they split into motor and sensory branches. This separation of the nerves bears risks of damaging both components of the nerves. However, this preparation does not only avoid misdirection of axons but will also conserve the function of the thenar nerve in such rare cases, where it was not affected by the injury. If the thenar branch is affected, we suggest performing the nerve transfer as an addition to an opponensplasty. When separating the SBUN and the DBUN, high efforts should be put in the preservation of the DBUN to either preserve its intact function or to keep it available as target for a transfer of the anterior interosseous nerve (Brown, Yee, et al., 2009; Wang & Zhu, 1997). If interchanging fibers between SBUN and DBUN appear very dense, a sural nerve graft can help to avoid nerve damage when branches are separated (Kozin, 2008).

Regeneration of axons from the donor across the nerve coaptation into the target is crucial for the result of sensory and motor nerve transfers. Most of our knowledge on how histomorphometric data correlate with clinical results of nerve transfers was gained through motor nerve transfers (Asaoka, Sawamura, Nagashima, & Fukushima, 1999; Boutros, Nath, Yüksel, Weinfeld, & Mackinnon, 1999). We assume that the methods of donor‐to‐target comparison may also be applied to sensory transfers. Commonly accepted methods to evaluate the results of nerve transfers are donor‐to‐target comparisons of histomorphometric nerve characteristics (Asaoka et al., 1999). Successful reinnervation is known to occur even when the donor is smaller than the target, because of collateral sprouting of axons in the proximal nerve stump (Jiang, Yin, Zhang, Fu, & Zhang, 2007; Totosy de Zepetnek, Zung, Erdebil, & Gordon, 1992). A commonly accepted threshold for successful motor nerve transfers is a donor‐to‐target axon ratio of 1:3 (Lutz et al., 2000).

We found axon ratios of 1: 1.1 for the SBRN to MN transfer and 1: 1:4 for the SBRN to SBUN transfer. Both ratios are better than the commonly accepted threshold. The axon density of the SBRN exceeds the MN and the SBUN (Table 1).

**Table 1:** Histomorphometric donor to target ratios

	SBRN: MN	SBRN: SBUN
Cross‐sectional fascicle area [mm²]	1: 2.0	1: 1.6
Axon number	1: 1.1	1: 1.4
Axon density [axons/mm^2^]	1: 0.7	1: 0.9

SBRN: superficial branch of the radial nerve; SBUN: superficial branch of the ulnar nerve; MN, median nerve.

Comparing donor‐to‐target cross‐sectional fascicle areas shows that the SBRN is an inferior donor. When comparing the absolute axon numbers, the difference is not as striking due to axon density, which reveals the SBRN as having a higher density than both the targets. The axon ratio is far below the commonly accepted threshold for successful nerve transfers of a 1: 3 ratio. From a histomorphometric perspective both nerve transfers can be expected to be successful.

The cross‐sectional areas we observed are in line with the clinical experience that the MN is larger than the SBUN, which is larger than the SBRN. The measured fascicle areas are smaller than in vivo due to volume loss at fixation, dehydration, and embedding of the specimen.

When harvesting the SBRN, the donor side defect, especially the loss of tactile sensibility of thumb and index finger may not be neglected (Bas & Kleinert, 1999). Some patients with sensory median defects grab small objects between the interphalangeal joint of the thumb and the metacarpophalangeal joint of the index, where the dorsal innervation provides some sensibility. If these areas want to be preserved from denervation, we suggest harvesting the SBRN distally to its bifurcation to preserve thumb and index branches. Unfortunately, we found the radial branches of the SBRN to be dominant in axon numbers (data not shown), which means that the amount of available axons in the ulnar branches are limited. To maintain a good donor‐to‐target ratio in these cases, we recommend to limit the size of the target as well and to select just some of the common digital nerves or parts of them. The ulnar aspect of the thumb should be prioritized followed by the radial aspect of the index and the ulnar aspect of the small finger.

The presented nerve transfers should be indicated in cases when direct suturing of the injured median or ulnar nerve was not possible or did not bring a satisfactory result.

Especially in proximal injuries, which are known for poor results, the nerve transfers are good treatment alternatives because, from a regenerative point of view, a proximal injury is converted to a distal injury.

For both transfers, careful evaluation of the overall hand function is crucial and in most cases conjoint procedures with other nerve or tendon transfers will bring the best solutions. We expect results to be best in young patients with intervention shortly after injury. Preoperatively, patients should be informed that tactile stimuli to the reanimated area will be associated to the area of the donor nerve in many cases. For means of protective sensibility, faulty representation of nerve areas is not a problem.

## 5 Conclusion

From our anatomic and histological data we conclude that the SBRN is a suitable donor for the MN and the SBUN. Our anatomic measurements show feasibility of the transfer and shall help to make a precise plan for an operation. The histomorphometric results reveal the SBRN as a sufficient donor. The macroscopically observed inferiority of the SBRN in size can be misleading in judging its quality as a donor. The high axon density of the SBRN partly outweighs its smaller cross‐sectional area. We consider the presented nerve transfers as promising treatment options to reanimate the sensibility of fingers and the thumb.

## Conflicts of Interest

None declared.
